# Subambient daytime radiative cooling to mitigate haze-induced amplification of urban heat islands

**DOI:** 10.1038/s41377-026-02391-6

**Published:** 2026-06-23

**Authors:** Minghao Dong, Qiuyu Chen, Zheng Zhang, Xiaodong Zhao, Peng Xiao, Zhen Chen

**Affiliations:** 1 Jiangsu Key Laboratory for Design and Manufacturing of Precision Medicine Equipment, School of Mechanical Engineering, Southeast University, Nanjing, China; 2 State Grid Jiangsu Electric Power Co. Ltd. Research Institute, Nanjing, China

**Keywords:** Green photonics, Metamaterials

## Abstract

High concentrations of particulate matter (PM) in cities intensify the urban heat island (UHI) effect by significantly altering the optical and thermophysical properties of the atmosphere. Although passive daytime radiative cooling technology (PDRC) offers a potential mitigation strategy, its performance under haze-polluted urban conditions remains largely unexplored. Here, we develop a general framework to model PDRC in the presence of haze pollution. Our analysis reveals that, due to the selective scattering of PM—which affects solar irradiation and infrared emission differently—the optimal photonic design for haze-polluted atmospheres should prioritize thermal emission within the atmospheric window while reducing emphasis on minimizing solar absorption. Guided by this principle, we optimized the thickness of a polydimethylsiloxane-based transparent thermal emitter by carefully balancing the tradeoff between solar absorption and thermal radiative emission. Experimental results highlight a critical shift: the performance advantage shifts from the thinner cooler under clear skies to the thicker design under haze-polluted conditions, which confirms our design strategy.

## Abstract (web summary)

We theoretically develop and experimentally demonstrate guidelines for photonic designs to enable subambient daytime radiative cooling under haze-polluted skies, which highlights selective scattering of sunlight and infrared thermal emission.

## Graphical abstract

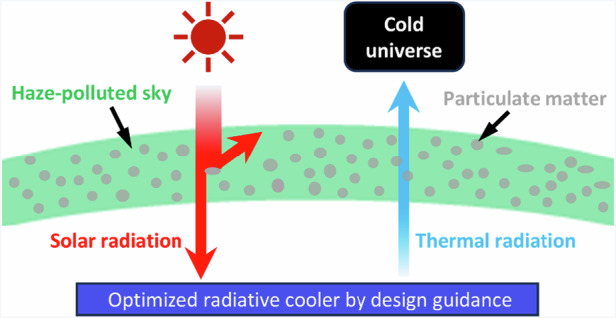

## Introduction

The urban heat island (UHI), a phenomenon of higher temperature in urban areas than in the surrounding rural areas, poses a threat to human health^1^. Cities are the primary source of anthropogenic aerosols^2^, resulting in elevated amounts of particulate matter (PM) in urban areas. As a result, haze pollution amplifies nocturnal UHI by approximately 0.7 K^3^, since haze has a great impact on the optical and thermophysical properties of the atmosphere^4^. More critically, without substantial human intervention, haze events are expected to become both more frequent and longer-lasting^5^.

Passive daytime radiative cooling (PDRC) technology expels radiative heat from terrestrial objects into the cold universe (3 K) through the atmospheric window (8-13 *µ*m)^6–8^ while reflecting sunlight, offering a sustainable solution to mitigate global warming. However, in pursuit of better cooling performance, PDRC research has focused predominantly on ideal weather conditions^9–11^, with far fewer studies dedicated to non-ideal scenarios like high-humidity environments^12–14^. In particular, PDRC in a haze-polluted urban atmosphere has not yet been systematically investigated. Correspondingly, the design strategy for radiative coolers tailored for haze conditions remains unclear.

Atmospheric PM is a complex mixture of organic and elemental carbon, ammonium, nitrates, sulfates, mineral dust, trace elements, and water^15^. Haze pollution directly impacts the climate through radiative absorption and scattering^16^. PM affects PDRC in two opposing ways: it attenuates solar radiation reaching the ground, which benefits cooling^17,18^, but it also degrades the transparency of the atmospheric window, thereby impairing the cooling effect^19^. Lacking a mathematical framework for quantitative analysis, the impact of haze on PDRC remains unclear, and photonic design consequently lacks clear guidance.

To address these shortcomings, we develop a general framework to quantitatively analyze the impact of haze on PDRC. Our analysis reveals that haze-induced attenuation of sunlight outweighs its effect on thermal radiation. Consequently, under haze-polluted skies, the design strategy should shift priority toward maximizing thermal emission within the atmospheric window, rather than focusing primarily on minimizing solar absorption—a significant departure from the approach under ideal conditions. As a numerical example to illustrate this design strategy, we theoretically optimized the thickness of the well-known PDRC material, a transparent polydimethylsiloxane (PDMS) film on Ag substrate, by resolving the tradeoff between solar absorption and thermal radiative emission. Experimental results reveal a clear contrast: while the thinner cooler outperforms its thicker counterpart under clear skies, this superiority reverses under haze-polluted conditions. These results underline the distinct design guidelines for the clear and the haze-polluted skies.

## Results

### Theoretical analysis

**Fig. 1:**
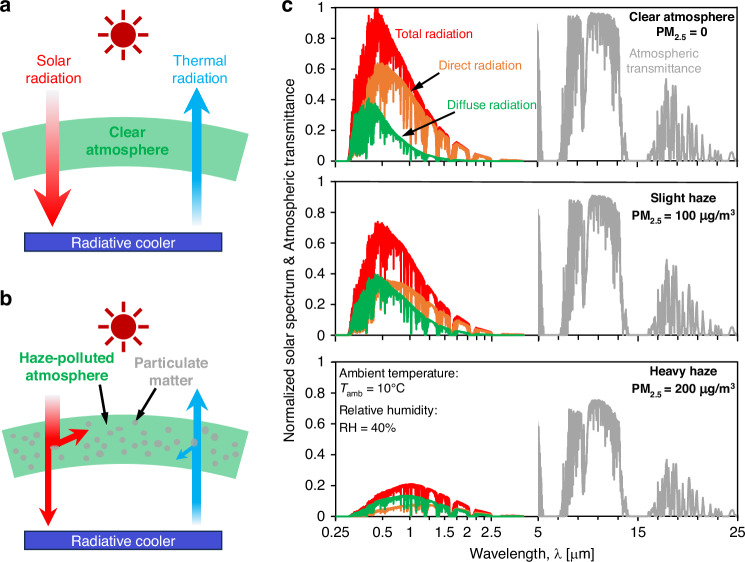
Explanation and justification of the effect of haze on radiative cooling. **a**, **b** Schematic of passive daytime radiative cooling (PDRC) under clear and haze-polluted skies. Particulate matter (PM) in a haze-polluted atmosphere scatters both sunlight and infrared thermal radiation from the radiative cooler. While the former is beneficial, the latter is detrimental to PDRC. **c** Solar spectrum and atmospheric transmittance at various haze concentrations, indicated with various PM_2.5_. With the increase of PM_2.5_, the attenuation of solar radiation is more significant than that of atmospheric transmittance. This selective scattering is the basis of the analysis in this work

Figure 1 depicts a schematic comparison of passive daytime radiative cooling (PDRC) under a clear sky (Fig. 1a) versus a haze-polluted sky (Fig. 1b). In the former, the radiative cooler absorbs solar radiation and exchanges infrared (IR) thermal radiation with the atmosphere and outer space. When the net thermal radiation surpasses the absorbed solar radiation, the cooler passively reduces its temperature (*T*_cooler_) below ambient temperature (*T*_amb_) until a steady state is achieved, leading to subambient daytime radiative cooling. In addition to the physics involved in the clear sky scenario (Fig. 1a), the haze-polluted case (Fig. 1b) adds new scatterings: the particulate matter (PM) scatters both incident sunlight and emitted infrared radiation. While the former intuitively aids PDRC and the latter hinders it, it is quantitatively non-obvious which effect is dominant. An even more crucial open question concerns the new design guidelines required for coolers operating under haze-polluted conditions.

To answer the open question above, we first quantify the influence of haze on solar radiation and the atmospheric transmittance that affects IR emission. Here we compute the solar radiation and the atmospheric transmittance using MODTRAN^20^ at various levels of haze pollution. As a concrete example, we fix *T*_amb_ = 10 °C and the relative humidity, RH = 40% (see Supplementary Fig. S10 for other combinations of *T*_amb_ and RH). Note here we choose to distinguish the level of haze pollution using the concentration of PM_2.5_^21^, and normalize the solar spectrum by the maximal intensity of the total solar radiance at PM_2.5_ = 0.

As shown in Fig. 1c, the total solar radiation, *G*_total_ (red), on the ground surface decreases with the increase of the haze concentration, as expected, since the PM scatters the sunlight. Note here *G*_total_ consists of two components^22^: direct radiation, *G*_direct_ (orange), received from the sun without having been scattered by the atmosphere, and diffuse radiation, *G*_diffuse_ (green), received from the sun after its direction has been changed by scattering. As the haze pollution intensifies, the reduction in *G*_direct_ surpasses that of *G*_diffuse_, because a part of *G*_direct_ is scattered into *G*_diffuse_ in this process. Similarly, atmospheric transmittance (gray) decreases as the haze pollution increases. It is worth noting that the decrease in solar radiation is more significant than the decrease in atmospheric transmittance. This selectivity, which is further justified in Supplementary Note S4 using Mie scattering theory, is the basis for new guidelines to design coolers under haze-polluted conditions.

Next, we figure out these new guidelines. To evaluate the performance of PDRC under varying weather conditions, the net cooling power of the cooler, with units of [W/m^2^], is given by

1 $${P}_{{\rm{cool}}}={P}_{{\rm{rad}}}\left({T}_{{\rm{cooler}}}\right)-{P}_{{\rm{atm}}}\left({T}_{{\rm{amb}}}\right)-{P}_{{\rm{solar}}}-h\left({T}_{{\rm{amb}}}-{T}_{{\rm{cooler}}}\right)$$

In this work, we fix the nonradiative heat transfer coefficient between the cooler and the environment, *h* = 8 W/(m^2^-K)^23,24^ (see Supplementary Fig. S11 for other *h*). In Eq. (1), the outgoing thermal radiation power from the cooler is

2 $${P}_{\mathrm{rad}}({T}_{\mathrm{cooler}})=\int {\mathrm{cos}}\uptheta \,{\rm{d}}\Omega {\int }_{0}^{\infty }{I}_{\mathrm{BB}}({T}_{\mathrm{cooler}},\uplambda )\upepsilon (\uplambda ,\uptheta ){\rm{d}}\uplambda$$

Here, $\int {\rm{d}}\Omega =2{\rm{\pi }}{\int }_{0}^{{\rm{\pi }}/2}\sin {\rm{\theta }}{\rm{d}}{\rm{\theta }}$ is the angular integral over a hemisphere. ${I}_{{\rm{BB}}}\left(T,{\rm{\lambda }}\right)=\frac{2{h}_{{\rm{c}}}{{\rm{c}}}^{2}}{{{\rm{\lambda }}}^{5}}\frac{1}{{{\rm{e}}}^{{h}_{{\rm{c}}}c/({\rm{\lambda }}{k}_{{\rm{B}}}T)}-1}$ is the spectral radiance of a blackbody at temperature *T*, where *h*_c_ is Planck’s constant, *k*_B_ is the Boltzmann constant, and *c* is the speed of light. ${\rm{\epsilon }}({\rm{\lambda }},{\rm{\theta }})$ is the spectral-directional emissivity of the radiative cooler. The downward atmospheric thermal radiation, including the contribution from PM, absorbed by the cooler is

3 $${P}_{\mathrm{atm}}({T}_{\mathrm{amb}})=\int {\mathrm{cos}}\uptheta\,{\rm{d}}\Omega{\int }_{0}^{\infty }{I}_{\mathrm{atm}}({C}_{\mathrm{haze}},{T}_{\mathrm{amb}},\uplambda ,\uptheta)\upalpha (\uplambda ,\uptheta ){\rm{d}}\uplambda$$

In Eq. (3), we utilize ${\rm{\alpha }}\left({\rm{\lambda }},{\rm{\theta }}\right)={\rm{\epsilon }}({\rm{\lambda }},{\rm{\theta }})$ in accordance with Kirchhoff’s radiation law. ${I}_{{\rm{atm}}}({C}_{{\rm{haze}}},{T}_{{\rm{amb}}},{\rm{\lambda }})$ is the downward spectral-directional radiance of the atmosphere related to the haze concentration (*C*_haze_), *T*_amb_ and RH. In this work, we correlate *C*_haze_ to PM_2,5_ for atmospheric spectra using commercial software, MODTRAN^20^, as shown in Fig. 1c (gray lines) and Fig. S5. The solar power absorbed by the cooler is

4 $${P}_{\mathrm{solar}}=\int {\mathrm{cos}}\uptheta \,{\rm{d}}\Omega {\int }_{0}^{\infty }{I}_{\mathrm{solar}}({C}_{\mathrm{haze}},\uplambda ,{\uptheta} )\upalpha (\uplambda ,\uptheta ){\rm{d}}\uplambda$$

Here, ${I}_{{\rm{solar}}}\left({C}_{{\rm{haze}}},{\rm{\lambda }},{\rm{\theta }}\right)$ is the spectral-directional solar radiance, including both direct and diffuse parts. Note here, ${I}_{{\rm{solar}}}$ also depends on the concentration of haze, *C*_haze_, which again can be correlated to PM_2,5_ using MODTRAN^20^ explicitly, as shown in Fig. 1c (colored lines). The maximal cooling power, *P*_max_, is obtained by enforcing *T*_cooler_ = *T*_amb_, and the temperature reduction (Δ*T* = *T*_amb_ − *T*_cooler_) is evaluated at *P*_cool_ = 0.

**Fig. 2:**
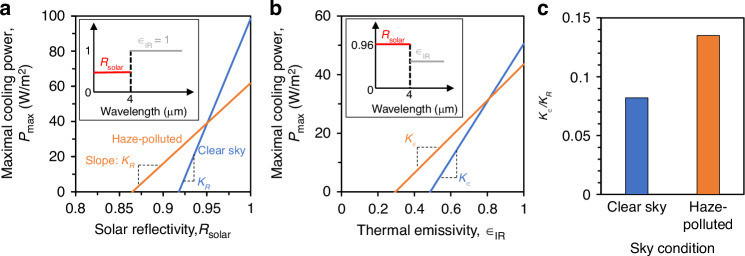
Theoretical analysis to contrast cooler design principles for haze-polluted versus clear skies. **a**, **b** Originating from the selective scattering of haze (Fig. 1c), under a haze-polluted sky (orange), sub-ambient cooling can be achieved with much lower solar reflectivity, *R*_solar_, or infrared (IR) emissivity, ϵ_IR_. In both cases, the slope, ${K}_{R}={\rm{d}}{P}_{\max }/{\rm{d}}{R}_{{\rm{solar}}}$ in (**a**) or ${K}_{{\rm{\epsilon }}}={\rm{d}}{P}_{\max }/{\rm{d}}{{\rm{\epsilon }}}_{{\rm{IR}}}$ in (**b**), is steeper under clear skies than that under haze-polluted skies. **c** The larger ratio, ${K}_{{\rm{\epsilon }}}/{K}_{R}$, in haze-polluted scenarios (orange) suggests a new guiding principle for cooler design

Using the mathematical framework established above, we now differentiate the design guidelines for radiative coolers under haze conditions from those under clear skies. In Fig. 2a, with the infrared emissivity fixed at ϵ_IR_ = 1, we compare the maximal cooling flux (*P*_max_) as a function of solar reflectivity (*R*_solar_), under clear (blue) and haze-polluted (orange) skies. A key finding is that the haze-polluted sky is more tolerant of designs with significantly lower *R*_solar_ to achieve sub-ambient cooling. For instance, whereas a minimum *R*_solar_ of 0.92 is required under clear skies, this requirement relaxes to *R*_solar_ = 0.87 under haze-polluted conditions. This contrast stems from the selective scattering of haze, as discussed in the context of Fig. 1c. On the other hand, as *R*_solar_ approaches unity, haze impacts cooling solely by degrading infrared emission. Consequently, in this high-*R*_solar_ limit (*R*_solar_ → 1), cooling performance under clear skies surpasses that under haze. Combining the low- and high-*R*_solar_ regimes, Fig. 2a reveals a much weaker dependence of *P*_max_ on *R*_solar_ for the haze-polluted case (orange), i.e., a smaller slope, ${K}_{R}={\rm{d}}{P}_{\max }/{\rm{d}}{R}_{{\rm{solar}}}$. Figure 2b shows a similar trend: the haze-polluted sky is more tolerant of designs with significantly lower ϵ_IR_ to achieve sub-ambient cooling. This is because, in the low-*R*_solar_ regime, for the same *R*_solar_, it absorbs much less solar radiation under the haze-polluted sky, and thus relatively smaller ϵ_IR_ is needed to keep the energy balance. Combining Fig. 2a, b, in Fig. 2c, we define a ratio, ${K}_{{\rm{\epsilon }}}/{K}_{R}$, to indicate the relative sensitivity of the cooling performance between the infrared and the solar regimes. Figure 2c suggests that for haze-polluted scenarios, one could relax the requirement on *R*_solar_ and focus on improving ϵ_IR_. Here we fix *T*_amb_ = 10 °C, RH = 40%, and PM_2.5_ = 200 *µ*g/m^3^ for the haze-polluted case. This quantitative result provides a distinct guiding principle for designing radiative coolers for haze-polluted environments.

### A numerical example

In this session, we use a proof-of-concept example to illustrate that the optimized photonic design for clear sky cannot be taken for granted for its application in haze-prone weather. In particular, to optimize a cooler for haze-polluted conditions, one should focus on increasing ϵ_IR_, even accepting a slight reduction in *R*_solar_, as suggested by the new guiding principles in Fig. 2.

**Fig. 3:**
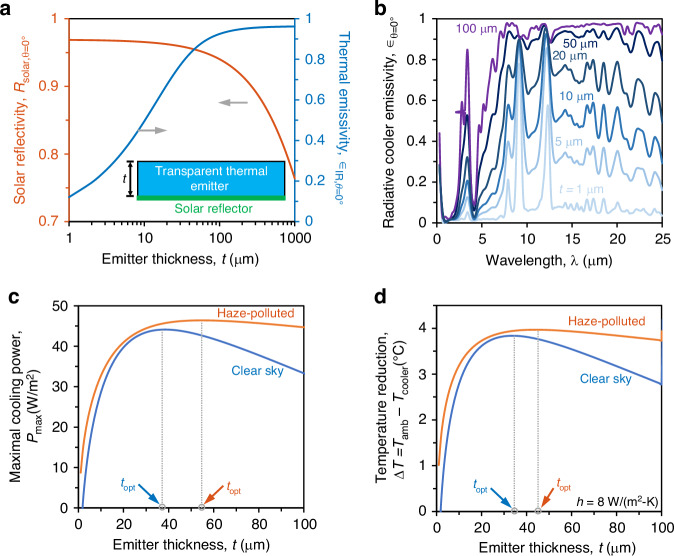
A numerical example to illustrate the guiding principles of designing coolers under a haze-polluted sky. **a** *R*_solar_ and ϵ_IR_ show opposite *t*-dependence. *R*_solar_ and ϵ_IR_ are calculated using Eqs. (5), (6). (**b**) Simulated spectral ϵ at different *t*. **c** Maximal cooling power, *P*_max_, and **d** temperature reduction, Δ*T*, as a function of *t*. This numerical example confirms our new guiding principles for photonic designs under haze-polluted skies, which are distinct from those under clear skies

We optimize a transparent thermal emitter, polydimethylsiloxane (PDMS)^25–28^, atop a silver reflector. Note here that the radiative cooler refers to the PDMS/Ag structure, while the emitter refers to the PDMS layer. In this structure, the silver substrate is used for reflecting sunlight, while the PDMS layer is for infrared emission, although it also affects *R*_solar_, since PDMS is not 100% transparent in the solar spectrum. Based on this argument, *R*_solar_ and ϵ_IR_ exhibit opposite trends as the increase of *t*, as confirmed in Fig. 3a. Note here the effective solar reflectivity and the effective thermal emissivity of the radiative cooler along the zenith angle are defined as

5 $${R}_{\mathrm{solar},{\rm{\theta }}=0^\circ }=\frac{{\int }_{{\rm{\lambda }}=0.3\mu {\rm{m}}}^{4\mu {\rm{m}}}{I}_{\mathrm{AM}1.5}\left({\rm{\lambda }}\right)\left[1-{\rm{\epsilon }}\left({\rm{\lambda }},{\rm{\theta }}=0^\circ \right)\right]{\rm{d}}{\rm{\lambda }}}{{\int }_{{\rm{\lambda }}=0.3\mu {\rm{m}}}^{4\mu {\rm{m}}}{I}_{\mathrm{AM}1.5}\left({\rm{\lambda }}\right){\rm{d}}{\rm{\lambda }}}$$

6 $${{\rm{\epsilon }}}_{\mathrm{IR},{\rm{\theta }}=0^\circ }=\frac{{\int }_{{\rm{\lambda }}=0.3\mu {\rm{m}}}^{25\mu {\rm{m}}}{I}_{\mathrm{BB}}\left(T=300{\rm{K}},{\rm{\lambda }}\right){\rm{\epsilon }}\left({\rm{\lambda }},{\rm{\theta }}=0^\circ \right){\rm{d}}{\rm{\lambda }}}{{\int }_{{\rm{\lambda }}=0.3\mu {\rm{m}}}^{25\mu {\rm{m}}}{I}_{\mathrm{BB}}\left(T=300{\rm{K}},{\rm{\lambda }}\right){\rm{d}}{\rm{\lambda }}}$$

Here, *I*_AM1.5_ is the AM1.5 solar spectrum (Fig. S4). *R*_solar,θ=0º_ and ϵ_IR,θ=0º_ (Fig. 3a) are calculated using Eqs. (5)-(6) and the spectral emissivity in Fig. 3b that is computed using the scattering-matrix method^29^. Note that for clarity, we present the atmospheric transmittance (Fig. 1c) and the cooler emissivity and reflectivity (Fig. 3a, b) along the zenith angle. In the calculation of cooling performance, we use the angle-independent properties.

The performance of PDRC is shown in Fig. 3c-d, in which we fix *T*_amb_ = 10 °C, RH = 40%, *h* = 8 W/(m^2^-K)^10,14^ for Δ*T*, and PM_2.5_ = 200 *µ*g/m^3^ for the haze-polluted case (see Supplementary Note S2 and Fig. S3 for the dependence of the cooling performance on the haze concentration). Three features of *P*_cool_ and Δ*T* are worth noting. First, both *P*_cool_ and Δ*T* under haze-polluted sky exceed those under clear skies. Since the designs are in the low-*R*_solar_ regime (left portion of the crossover in Fig. 2), the impact of PM on solar radiation surpasses its impact on atmospheric transmittance, as shown in Fig. 1c. Second, there exists an optimal thickness, *t*_opt_, regardless of the weather conditions. This optimal value is a direct consequence of the competing effects between the decrease of *R*_solar_ and the increase of ϵ_IR_ as *t* increases. In the low-*t* regime, the increase of IR emission dominates, and thus both *P*_cool_ and Δ*T* increase with the increase of *t*. In the high-*t* regime, however, ϵ_IR_ saturates at 100%, and thus the decrease of *R*_solar_ becomes predominant, resulting in a decrease in *P*_cool_ and Δ*T* as *t* increases (see Supplementary Note S3 and Fig. S7 for more details). Third, *t*_opt_ under a haze-polluted sky is larger than that under a clear sky, since a radiative cooler with higher ϵ_IR_ is favored in the haze-polluted scenario, in which condition the decrease in solar radiation is more significant than the decrease of atmospheric transmittance, as shown in Fig. 1c. A sensitive analysis of the cooling performance is illustrated in Fig. S8. Complementary analysis for an ultra-humid scenario is presented in Fig. S16, which, together with Fig. 3, reinforces the guiding principle of designing radiative coolers under haze-polluted skies as shown in Fig. 2.

### Experiments

**Fig. 4:**
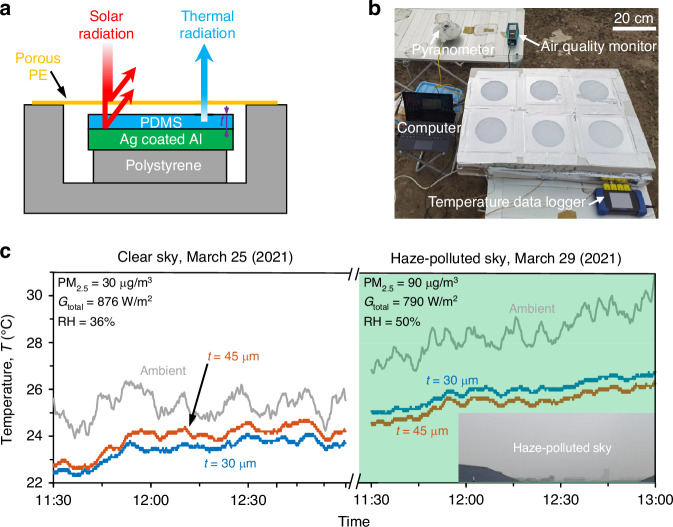
Experimental verification of the different guiding principles of photonic designs for PDRC under clear and haze-polluted skies. **a**, **b** Schematic and photograph of the experimental setup. A silver-coated aluminum plate, beneath the transparent PDMS film, serves as a solar reflector. Porous polyethylene (PE) film is employed to achieve subambient cooling in Nanjing, a very humid city in China. **c** Contrast cooling performance between the two coolers optimized for the clear and the haze-polluted skies: the thinner (thicker) cooler performs better under clear (haze-polluted) sky, consistent with the new guiding principles in Fig. 2 and the theoretical prediction in Fig. 3c

**Fig. 5:**
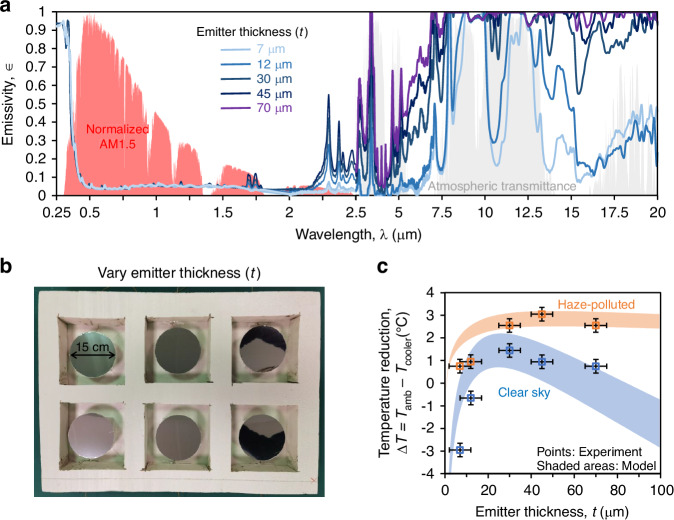
Experimental verification of the optimization. **a** Measured emissivity spectra of radiative coolers with 5 different emitter thicknesses: 7, 12, 30, 45 and 70 *µ*m. The normalized solar spectrum AM1.5 (red) and atmospheric transmittance (gray) are for reference. **b** Photograph of the radiative coolers. **c** Five simultaneous measurements at noon, corresponding to the peak solar intensity of the day, under a clear sky (blue points; Mar. 25, 2021: PM_2.5_ = 30 *µ*g/m^3^) and a haze-polluted sky (orange points; Mar. 29, 2021: PM_2.5_ = 90 *µ*g/m^3^), respectively

We conduct a series of experiments to validate our designs above. Figure 4a schematically describes the basic experimental setup. To minimize thermal interaction between the radiative cooler and the surroundings, we construct an enclosure with the bottom and side walls composed of polystyrene, which are then coated with aluminized mylar (Fig. 4b). The experiments are conducted in Nanjing, China. To achieve subambient daytime cooling in such a humid region^13,14^, a 25-*µ*m-thick layer of porous polyethylene (PE), with low solar transmittance and high MIR transmittance^30–33^, is used to seal the top of the enclosure to mitigate solar heating and convection, while maintaining unobstructed thermal coupling between the radiative cooler and outer space. The spectrum of the porous PE is shown in Fig. S1. Note that in the theoretical model in Fig. 5c, we include the effect of the porous PE (see Supplementary Note S1 and Fig. S2 for more details).

Two radiative coolers with different thicknesses, *t* = 30 *µ*m and 45 *µ*m, respectively, of transparent emitters are fabricated. Using experimentally measured spectra, we obtain the effective *R*_solar_ and the effective ϵ_IR_: *R*_solar_ = 95% and ϵ_IR_ = 67% for the thinner cooler, and *R*_solar_ = 94% and ϵ_IR_ = 75% for the thicker one. With these two coolers, we conduct cooling experiments on a rooftop at Southeast University, Nanjing, China. Local weather conditions are obtained from a weather station at Nanjing^34^. The inset photo of Fig. 4c shows the haze-polluted sky.

Figure 4c presents the experimental results of subambient PDRC. Under a clear sky (left panel), the temperature of the thinner cooler (blue) is lower than that of the thicker one (orange). Under haze-polluted conditions (right panel), two features are worth noting. First, the temperature reduction (Δ*T* = *T*_amb_ − *T*_emitter_) exceeds that under clear skies, regardless of the thickness of the emitter. This result is consistent with the prediction in the low-*R*_solar_ (Fig. 2a) and the low-ϵ_IR_ (Fig. 2b) regimes. Second, the thicker cooler (orange) reaches Δ*T* = 3.1 °C, surpassing the performance of the thinner cooler (blue), which shows an opposite trend to that under a clear sky (left panel). Both features manifest the selective scattering of the particulate matter of the haze-polluted sky to sunlight (see Supplementary Note S4 and Fig. S9 for more details), and thus confirm our guideline to design coolers for haze-polluted scenarios, which is distinct from that for clear skies (Figs. 2–3). In Supplementary Note S5, we combine theoretical arguments (Fig. S15 and Table S1) and additional measurements (Fig. S14) to confirm that the contrast shown in Fig. 4c is dominated by haze.

We next validate our optimization results. We prepare a set of radiative coolers with various thicknesses of transparent thermal emitter: 7 *µ*m, 12 *µ*m, 30 *µ*m, 45 *µ*m, and 70 *µ*m, along with an additional reference cooler, whose spectra (Fig. 5a) are measured using a UV-VIS-NIR spectrophotometer and a Fourier transform infrared (FTIR) spectrometer with integrating spheres. Six radiative coolers, each with a diameter of 15 cm, are secured into six chambers and separated by 5 cm of polystyrene insulation (Fig. 5b). The differences in sample appearance with differing thickness are due to the illumination conditions. Figure 5c shows representative experimental results of a clear-sky scenario (PM_2.5_ = 30 *µ*g/m^3^) and the haze-polluted case (PM_2.5_ = 90 *µ*g/m^3^), respectively. The optimized emitter (*t* = 45 *µ*m) for the haze-polluted weather achieves radiative cooling of 3.1 °C below ambient. The horizontal error bars originate from the accuracy of polymer thickness measurements, while the vertical error bars stem from the uncertainty of the temperature data logger and the thermocouple. These experiments (points) are consistent with our theoretical model predictions (shaded areas) guided by the new principles of photonic designs under haze-polluted skies. More experimental results are shown in Figs. S12-S14.

## Discussion

We have developed a general framework to describe radiative cooling under haze-polluted skies. In contrast to the clear sky, the elevated concentration of particulate matter (PM) in haze-polluted scenarios not only scatters sunlight but also degrades the transmittance of the atmospheric transparency window. Our model indicates that the scattering effects of haze on sunlight greatly surpass its weakening effects on the atmospheric window. Therefore, compared to the clear-sky case, the photonic design for haze-polluted conditions should shift priority toward maximizing infrared emission through the atmospheric transparency window, even at the expense of some compromise in solar reflectivity.

We end by discussing the optimized design of various typical polymer-based PDRC materials under haze-polluted conditions (see details in Supplementary Fig. S6), with the following remarks: First, the optimized structures could be employed long-term in cities severely impacted by haze pollution, such as those in India^35^, which endure year-round haze contamination. Second, in areas with lighter haze pollution, the optimized structures ought to be replaced seasonally, as haze predominantly occurs in winter^36^. Last but not least, a dynamic spectrum scheme^37^, either actively or passively regulated, would be ideal for switching between haze-polluted and clear weather conditions.

## Materials and methods

### Fabrication of transparent emitters

A Sylgard 184 (Dow Corning) mixture is prepared by blending the base elastomer and the curing agent at a 10:1 weight ratio. The mixture is stirred for five minutes and then degassed in a vacuum chamber for 30 minutes until no bubbles remain. For each sample, the degassed PDMS pre-polymer is dispensed onto the center of the commercial silver-coated aluminum plate (Alanod, 4400AG) with a diameter of 15 cm. The spin-coating cycle consists of two stages: First, a constant initial spin is at 500 rpm for ten seconds to ensure uniform coverage of the substrate. Next, the spin speed is varied to control the final film thickness. The coated substrates are subsequently transferred to a leveled hotplate and cured at 80 °C for two hours to form solid, cross-linked PDMS films as transparent emitters.

### Measurements

UV-VIS-NIR transmission and reflection spectra are measured with a spectrophotometer (Agilent, Cary 6000i) equipped with tungsten halogen and deuterium lamps. A diffuse reflectance accessory (integrating sphere; Agilent) and a variable-angle specular reflectance accessory (Agilent) are applied for comprehensive reflection property measurements. MIR transmission and reflection spectra are measured using a Fourier transform infrared (FTIR) spectrometer (Thermo Fisher Scientific, Nicolet iS50) equipped with an integrating sphere (PIKE, Mid-IR IntegratIR) and a variable specular reflection accessory (PIKE, VEEMAX III). Integrating spheres are used to measure the scattered light from the full solid angle in both the solar and the MIR regions. The thicknesses of polymers are measured using a coating thickness gauge (SMART SENOR, AR932D).

The concentration of PM_2.5_ is measured and recorded with an air quality monitor (BLATN, BR-SMART). Temperatures are measured using K-type thermocouples (Omega, TT-K-30-SLE), and a temperature data logger (Omega, RDXL6SD) is utilized for real-time temperature acquisition. Solar irradiance power is measured with a pyranometer (KIPP & ZONEN, CMP22), and a data logger (KIPP & ZONEN, METEON) is employed to capture real-time data. The humidity is recorded using a humidity recorder (Jingchuang, GSP-6). The ambient temperature and relative humidity are measured in a Stevenson screen situated 1.5 meters above ground level.

## Supplementary information

Supplementary Information for Subambient Daytime Radiative Cooling to Mitigate Haze-Induced Amplification of Urban Heat Islands

## Data Availability

The authors declare that all data supporting the findings of this study can be found within the paper and its Supplementary Information files. Additional data supporting the findings of this study are available from the corresponding author (Z.C.) upon reasonable request.
