# Supplementary material for: Subambient daytime radiative cooling to mitigate haze-induced amplification of urban heat islands
Source: Light Sci Appl. 2026 Jun 23;15:278. doi: 10.1038/s41377-026-02391-6 (PMC13291325; doi:10.1038/s41377-026-02391-6)
Supplement: Supplementary file 1 — Supplementary Information for Subambient Daytime Radiative Cooling to Mitigate Haze-Induced Amplification of Urban Heat Islands [file 41377_2026_2391_MOESM1_ESM.pdf]

Supplementary Information

Subambient daytime radiative cooling to mitigate haze-induced amplification of urban heat islands

Minghao Dong[1], Qiuyu Chen[1], Zheng Zhang[1], Xiaodong Zhao[1], Peng Xiao[2,*] and Zhen Chen[1,*]

[1]Jiangsu Key Laboratory for Design and Manufacturing of Precision Medicine Equipment, School of Mechanical Engineering, Southeast University, Nanjing 211189, China
[2]State Grid Jiangsu Electric Power Co., Ltd. Research Institute, Nanjing 211103, China
*Correspondence: zhenchen@seu.edu.cn; vodoco@foxmail.com

Supplementary figures:

Figure S1: Spectrum of the porous polyethylene.
Figure S2: Thermal circuit of the experimental setup.
Figure S3: Daytime radiative cooling performance as a function of haze concentration.
Figure S4: AM1.5 solar spectrum.
Figure S5: Atmospheric radiance at different haze concentrations.
Figure S6: Optimization of various polymers.
Figure S7: Power as a function of emitter thickness.
Figure S8: Sensitive analysis with a variation in haze concentration.
Figure S9: Scattering efficiency of the haze particles.
Figure S10: Solar spectrum and atmospheric transmittance under various ambient conditions.
Figure S11: Cooling performance with varying nonradiative heat transfer coefficients.
Figure S12: Additional experimental results regarding Fig. 4c of the main text.
Figure S13: Additional experimental results regarding Fig. 5c of the main text.
Figure S14: Additional experimental results of Fig. 4c of the main text.
Figure S15: Contributions of atmospheric compositions to downward radiation.
Figure S16: Cooling performance in humid environments.

Supplementary table:

Table S1: Meteorological data used for calculation.

Supplementary notes:

Note S1: Comparison between theoretical model predictions and experimental results.
Note S2: Daytime radiative cooling at varying haze concentration.
Note S3: Analysis of various components of the power as a function of the cooler thickness.
Note S4: Scattering efficiency of haze particles.
Note S5: Detailed analysis of the experimental results in Fig. 4c.

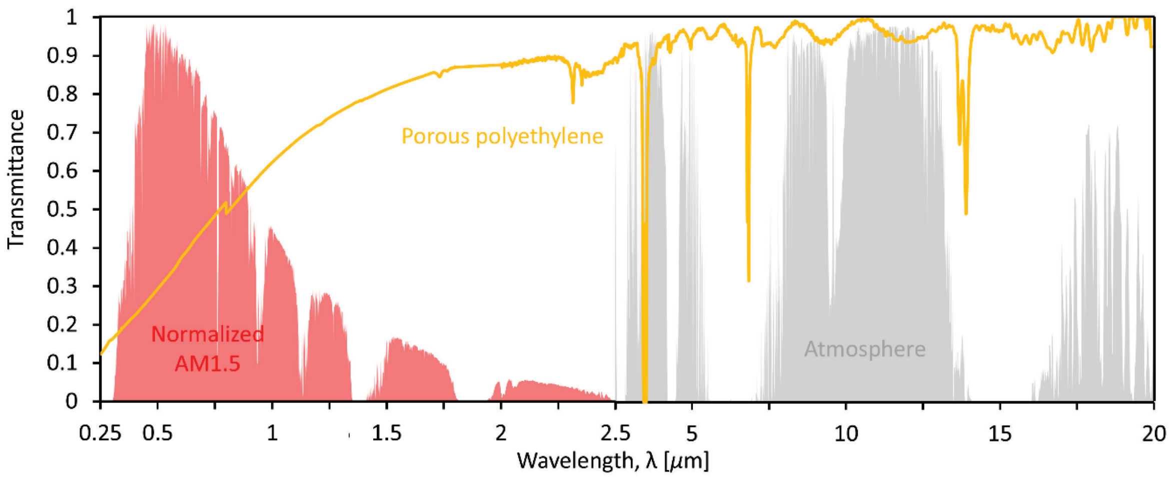

Figure S1. Spectrum of the porous polyethylene (PE). The PE film, with relatively low solar transmittance and high MIR transmittance within the atmospheric window (8-13 μm), is employed to block sunlight and reduce the parasitic heat by air convection, while permitting thermal radiation to permeate. Normalized AM1.5 solar spectrum (red) and atmospheric transmittance (gray) are shown for reference.

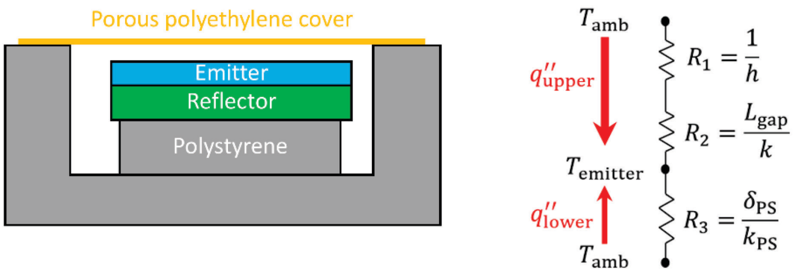

Figure S2. Schematic of the enclosure used in our experiments, and the corresponding thermal circuit to analyze the parasitic heat loss in the experimental setup. Here we neglect the conduction through the side walls of the enclosure because of its small cross section area and low thermal conductivity.

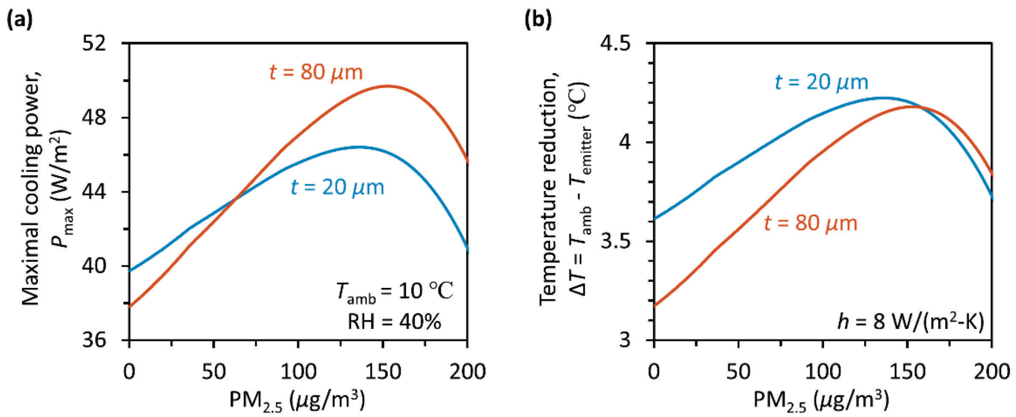

Figure S3. Performance of passive daytime radiative cooling (PDRC). (a) Maximal radiative cooling power (P_{max}) and (b) temperature reduction ($\Delta T = T_{amb} - T_{cooler}$), as a function of haze ($PM_{2.5}$) concentration. Here we fix other environmental conditions at an ambient temperature (T_{amb}) of 10 ℃ and a relative humidity (RH) of 40%.

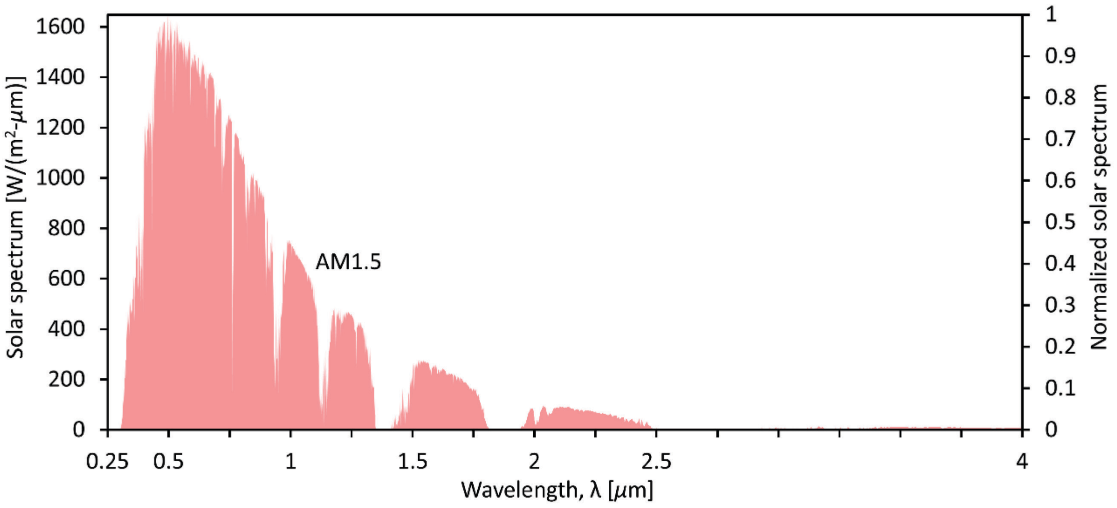

Figure S4. AM1.5 solar spectrum. The normalized solar spectrum is defined as $I_{\text{normalized}} = \frac{I}{I_{\text{max}}}$, where I_{max} is the maximal intensity. We use this standard spectrum to calculate the effective solar reflectivity in equation (5) of the main text.

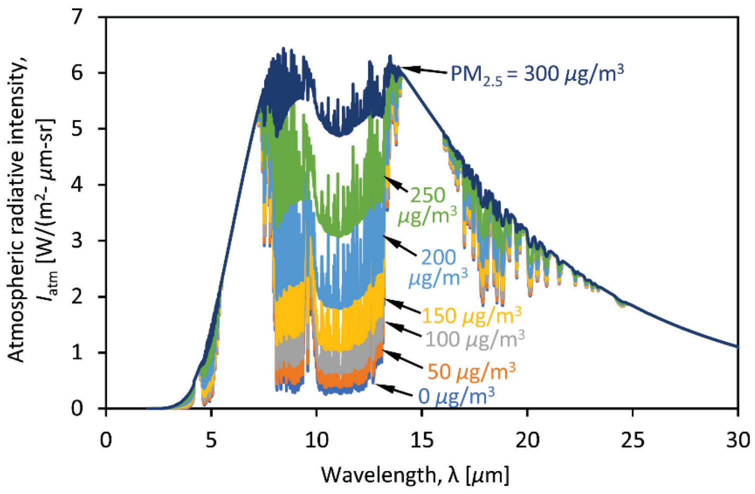

Figure S5. Atmospheric radiative intensity, I_{atm}, calculated using Modtran at different haze concentrations. Here we show I_{atm} only along the zenith angle for clarity. Other environmental conditions are fixed at an ambient temperature of 10 ℃ and a relative humidity of 40%.

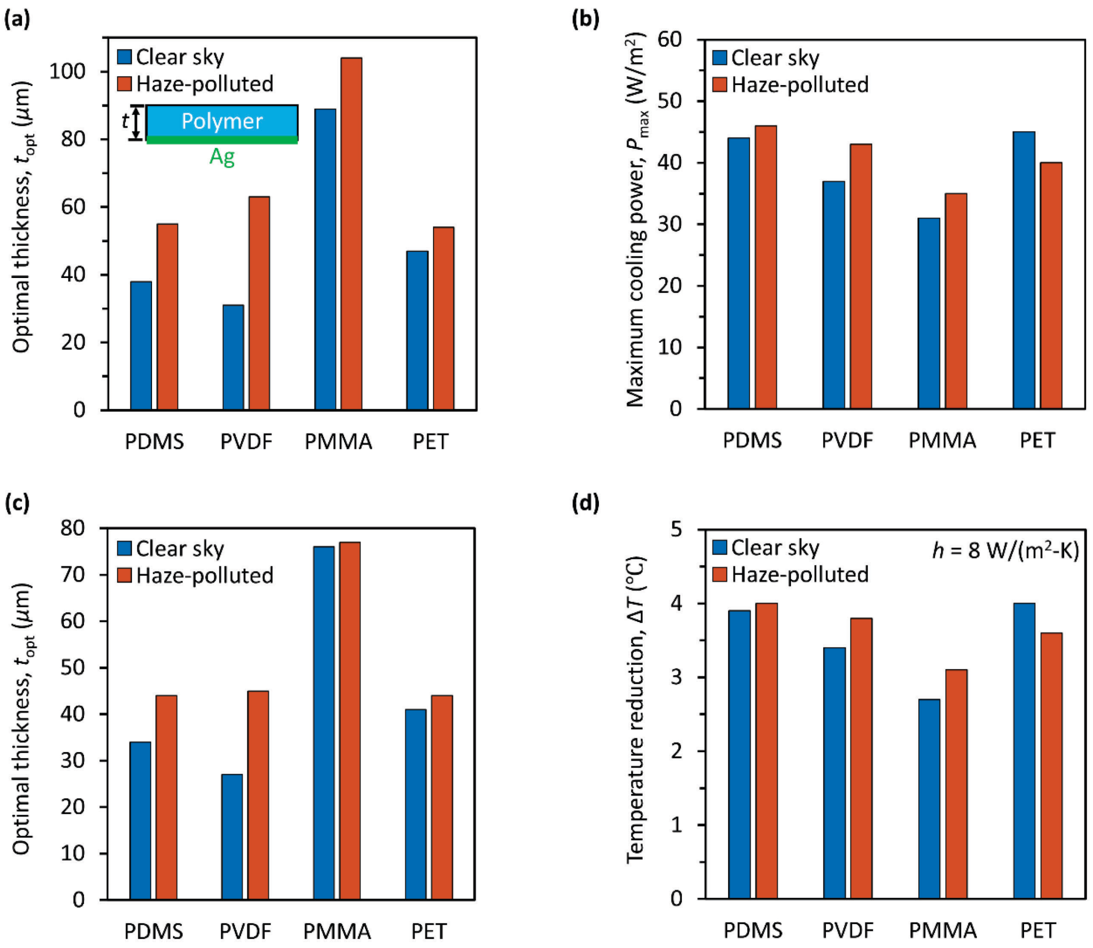

Figure S6. Optimization of various polymers. (a) Optimal thicknesses of polymers for maximum cooling power. (b) Maximum cooling power using the polymer at the optimal thickness. (c) Optimal thickness of polymers for maximum temperature reduction. (d) Temperature reduction (ΔT) using the polymer at the optimal thickness. These optimizations reveal the same shift: the cooling performance advantage shifts from the thinner polymer under clear skies to the thicker polymer under haze-polluted conditions. These results are calculated at an ambient temperature of 10 °C, a relative humidity of 40%, a nonradiative heat transfer coefficient (h) of 8 W/(m^2-K) for ΔT, and a $PM_{2.5}$ concentration of 200 μg/m^3 for the haze-polluted condition.

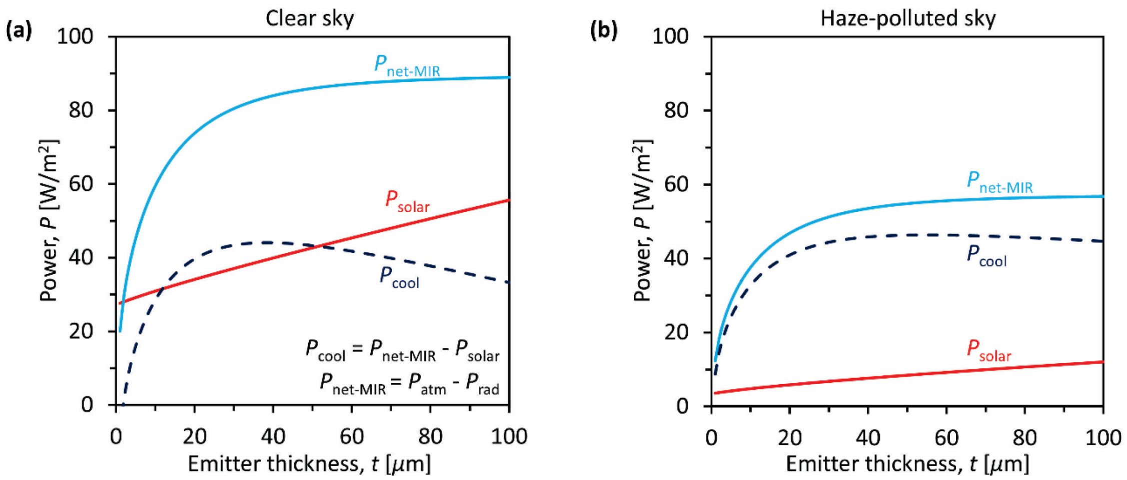

Figure S7. Solar absorption power (P_{solar}), net MIR power ($P_{net-MIR}$), and net cooling power (P_{cool}) as a function of transparent emitter thickness (t) in (a) clear atmosphere and (b) haze-polluted atmosphere. As t increases, both P_{solar} and $P_{net-MIR}$ ascend, while P_{cool} initially rises and thereafter diminishes. Compared to a clear scenario, the presence of haze leads to a reduction in both P_{solar} and $P_{net-MIR}$, with the diminution of P_{solar} surpassing that of $P_{net-MIR}$. Consequently, the optimal emitter thickness (t_{opt}) under haze-polluted conditions exceeds that in clear conditions.

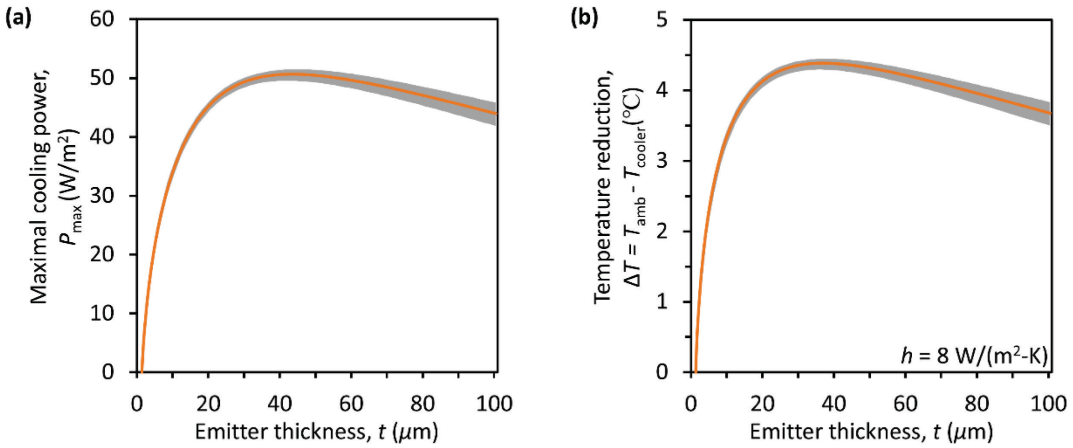

Figure S8. Sensitive analysis for (a) maximal cooling power and (b) temperature reduction with a ±20% variation in $PM_{2.5}$ concentration. The orange lines represent the cooling performance at a typical $PM_{2.5}$ = 100 μg/m^3. The grey shaded areas denote the cooling performance within a $PM_{2.5}$ concentration range of 100±20 μg/m^3. Here we fix other environmental conditions at an ambient temperature (T_{amb}) of 10 °C and a relative humidity of 40%.

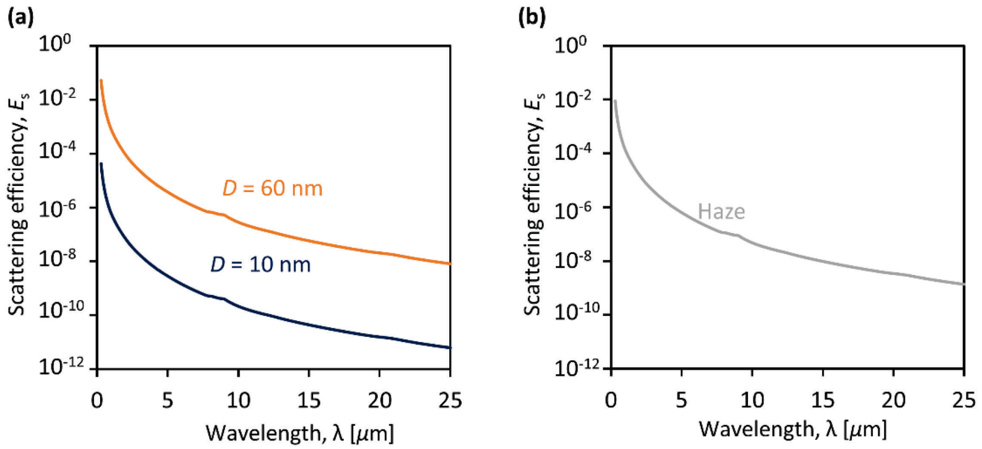

Figure S9. (a) Scattering efficiency of particles with diameters of 10 nm and 60 nm, respectively. The particles of around D = 10 nm and 60 nm account for 75 % and 15% of aerosols, respectively [1]. (b) Scattering efficiency of haze particles. Here we use the average optical constants of carbon and silica as the effective optical constants of haze particles.

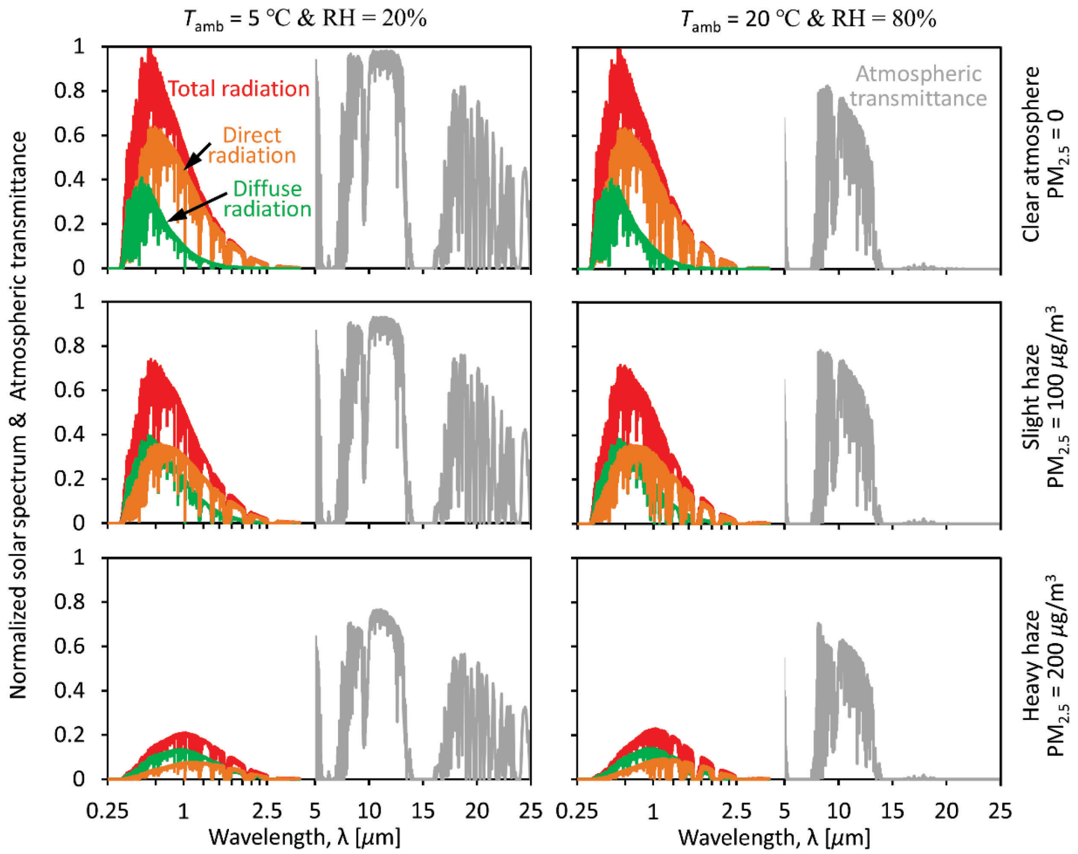

Figure S10. Solar spectrum and atmospheric transmittance under various ambient conditions. There are three features worth noting. First, similar to Fig. 1(a) of the main text, the diminishing effect of haze on solar radiance surpasses its impact on the atmospheric window. Second, while the variations in the solar spectrum at varying ambient temperatures (T_{amb}) and relative humidity (RH) are minimal, the differences in the atmospheric windows, especially the major window (8-13 μm) and the second window (16-25 μm) [2], are substantial. That is because the atmospheric window transparency is strongly related to the precipitable water vapor (PWV), which correlates with the combination of T_{amb} and RH [3]. Last, the weakening effect of haze on the major atmospheric window surpasses its impact on the second atmospheric window, as haze selectively scatters thermal radiation (Fig. S9).

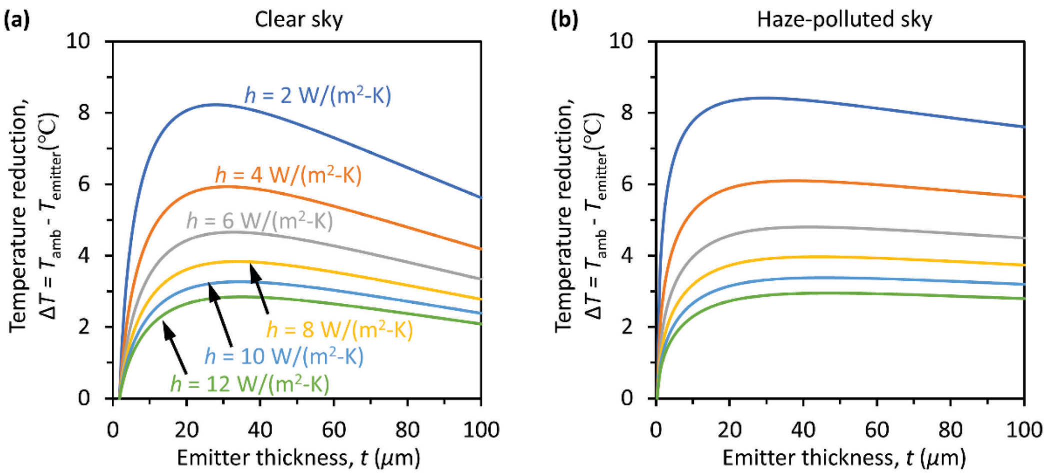

Figure S11. Temperature reduction, $\Delta T = T_{\mathrm{amb}} - T_{\mathrm{emitter}}$, as a function of transparent emitter thickness (t) with varying nonradiative heat transfer coefficients (h) in (a) a clear atmosphere and (b) a haze-polluted atmosphere at $PM_{2.5}$ = 200 μg/m^3. The radiative cooler consists of a top PDMS layer and a silver substrate. Other conditions are ambient temperature (T_{amb}) of 10 °C and relative humidity (RH) of 40%.

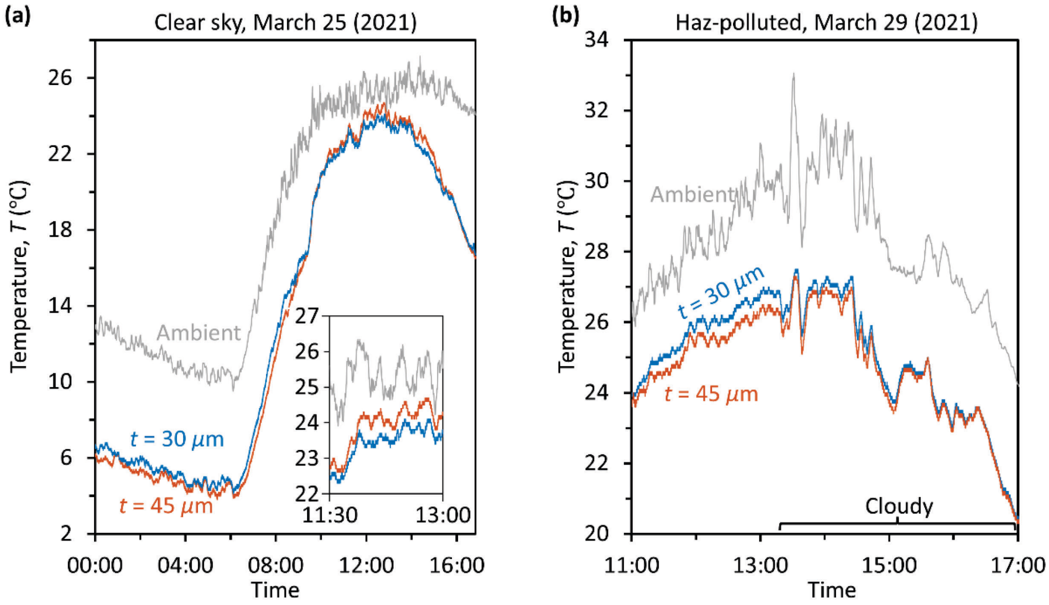

Figure S12. More experimental results regarding Fig. 4c of the main text. (a) In a clear sky, the thicker cooler temperature is first lower than that of the thinner one at night, as the thicker cooler exhibits higher thermal emission. At noon, the thinner cooler exhibits superior performance due to its elevated solar reflection. (b) Under haze pollution, the performance of the thicker cooler surpasses that of the thinner one, even at noon, due to the increased scattering of solar irradiance. Clouds began to appear at approximately 13:20, diminishing the performance difference between the two coolers.

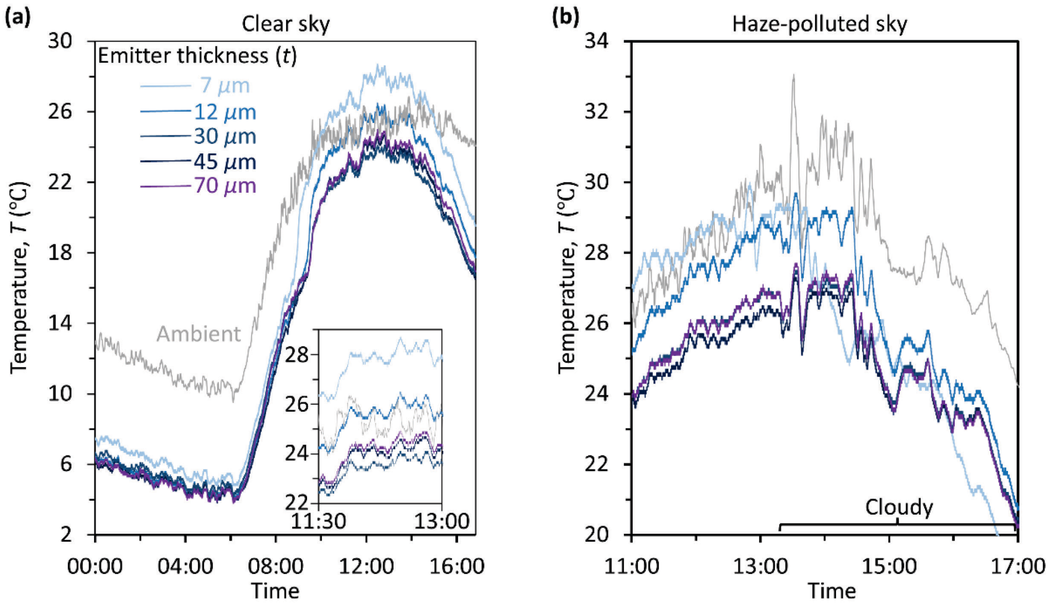

Figure S13. Supplementary experimental results to Fig. 5c of the main text. (a) Under clear skies, the 30 μm thick cooler performs the best at daytime. (b) Under haze-polluted skies, the t = 45 μm cooler exhibits the best cooling performance. Note that after ~13:30, the presence of clouds diminished the temperature differences among these coolers. The comparison between (a) and (b) shows that the optimal thickness moves from a thinner emitter under clear skies to a thicker one under haze-polluted conditions.

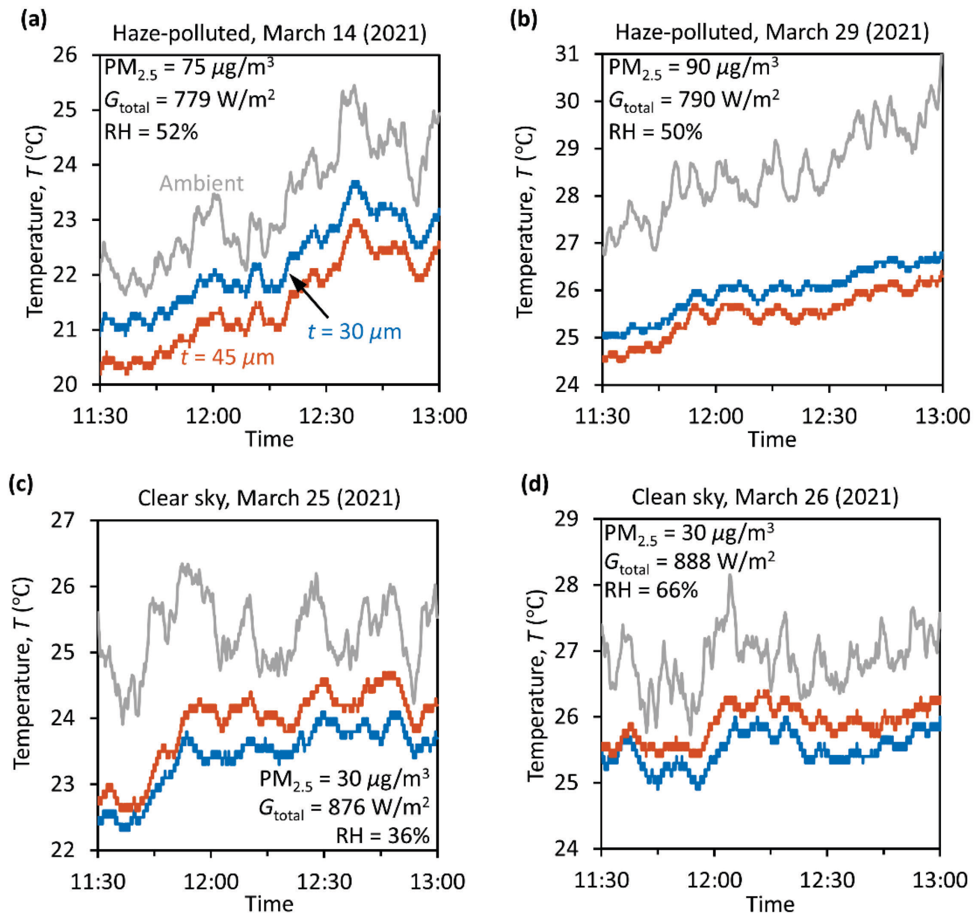

Figure S14. Experimental results with 2 different emitter thicknesses (t): 30 μm and 45 μm, under various atmospheric conditions. (a-b) Under haze pollution, the thicker cooler is preferable. (c-d) In a clear sky, the thinner cooler is advantageous.

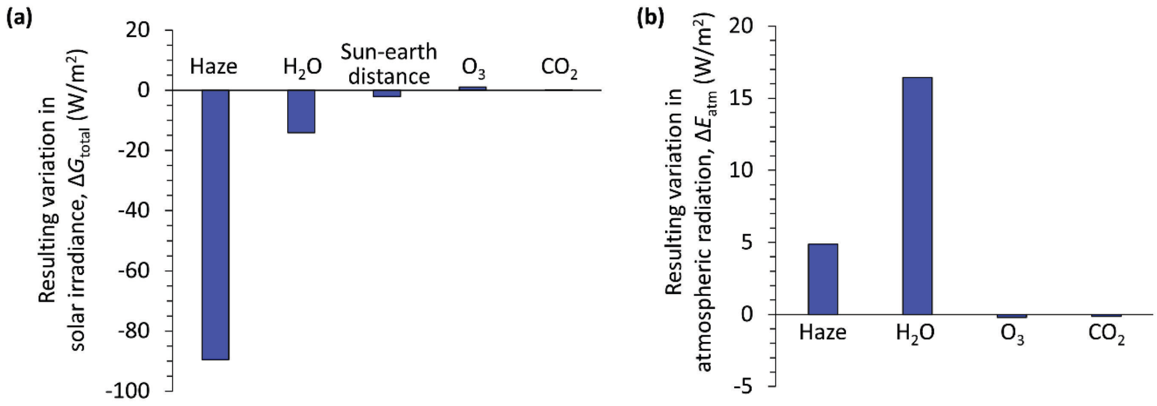

Figure S15. Contributions of every atmospheric composite to the variation in solar irradiance, ΔG_{total}, and atmospheric radiation, ΔE_{atm}, between March 25 and March 29, 2021. While haze is predominant in ΔG_{total}, water vapor is more significant for ΔE_{atm}. The contributions of other compositions are negligible. The meteorological data are listed in Table S1.

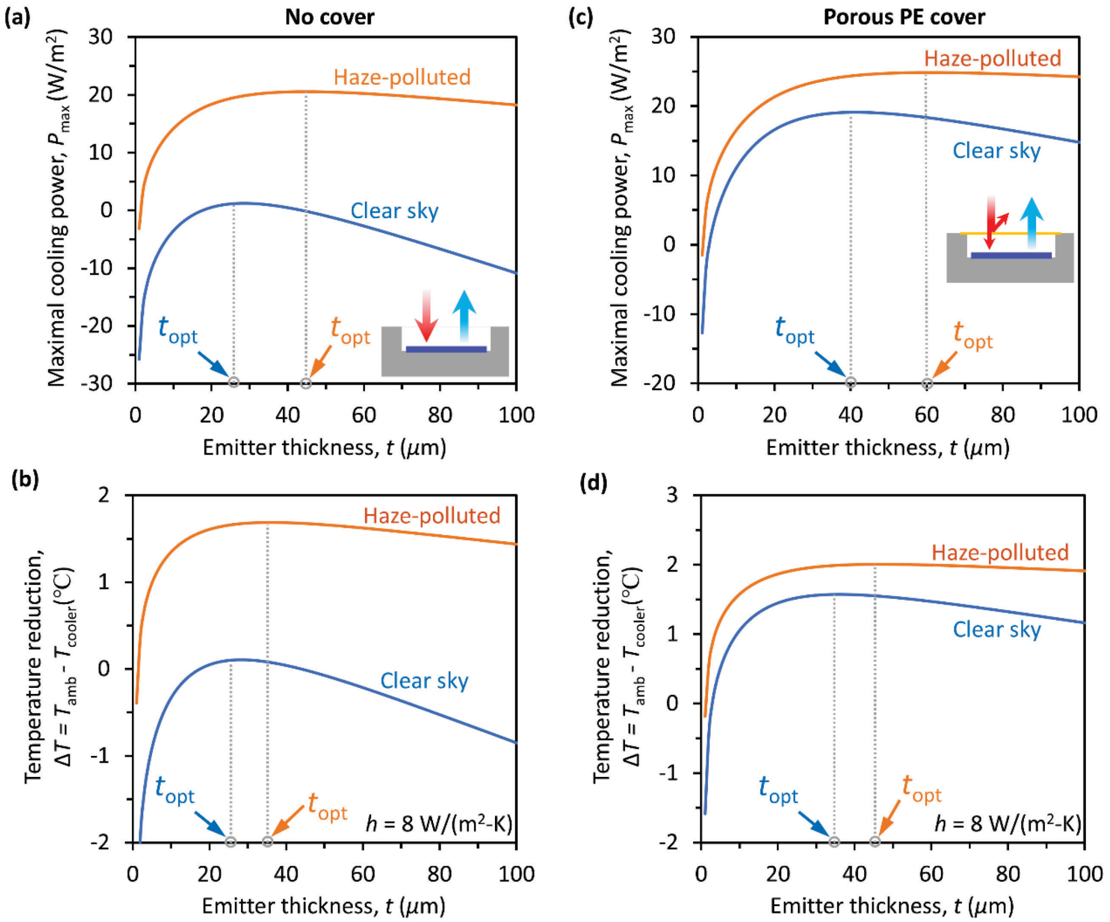

Figure S16. Complimentary analysis to Fig. 3 of the main text to demonstrate our design guidance for an ultra-humid scenario at T_{amb} = 25 °C and RH = 100%. Cooling performance without (left column) and with (right column) the porous PE cover. Similar to that under a dry climate (Figs. 3c-d of the main text), the optimization shifts from the thinner emitter under clear skies (blue lines) to the thicker emitter under haze-polluted conditions (orange lines). Here, we consider the effect of the cover on the radiation transfer but neglect its effect on the nonradiative heat transfer. Again. we fix the nonradiative heat transfer coefficient, h = 8 W/(m^2-K) for the calculation of temperature reduction (ΔT), and a $PM_{2.5}$ concentration of 200 μg/m^3 for the haze-polluted condition.

Table S1. Meteorological data, obtained from the China Meteorological Data Service Centre [4], to calculate the variations on solar irradiance, ΔG_{total}, and atmospheric radiation, ΔE_{atm}, from March 25 to March 29, 2021.

	Date		Resulting variation	
**Variable**	**25-Mar-2021 (Clear sky)**	**29-Mar-2021 (Haze-polluted)**	**ΔG_{solar} (W/m^2)**	**ΔE_{atm} (W/m2)**
**Haze**	30 μg/m^3	90 μg/m^3	-89	5
**PWV (H_2O)**	1.7 cm	2.4 cm	-14	16
**Sun-earth distance**	-	-	-2	-
**O_3**	98 μg/m^3	89 μg/m^3	1.0	-0.2
**CO_2**	285 ppm	276 ppm	0.05	-0.1

Supplementary Note S1: Comparison between model predictions and experimental results

For a fair comparison to the experimental results in Fig. 5 of the main text, we need to make some key modifications to the model for practical experimental scenarios, including the optical properties of the porous polyethylene (PE) cover and the estimate of the range of the nonradiative heat transfer coefficient (h).

Firstly, we take into account the optical properties of the porous PE cover (Fig. S1), which modifies the emissivity spectrum of the emitter, $\epsilon(\lambda, \theta)$, of the main text to

$$\epsilon'(\lambda, \theta) = \epsilon(\lambda, \theta) \times \frac{\tau_{\mathrm{PE}}(\lambda, \theta) + \alpha_{\mathrm{PE}}(\lambda, \theta)}{1 + r_{\mathrm{PE}}(\lambda, \theta) \times [\epsilon(\lambda, \theta) - 1]} \tag{S1}$$

In equation (S1), α_{PE}, τ_{PE} and r_{PE} are the absorptivity, transmissivity and reflectivity of the porous PE cover, respectively, which are characterized using the Fourier transform infrared spectroscopy (FTIR).

At last, we estimate h using the thermal circuit as shown in Fig. S2: the upper path couples the emitter to the environment through convection and air conduction above and below the porous polyethylene (PE) cover, and the lower path through the polystyrene bottom. Here, we neglect the conduction through the sidewalls of the enclosure because of its small cross-sectional area. The corresponding thermal resistors are

$$R_1 = \frac{1}{h} \tag{S2}$$

$$R_2 = \frac{L_{\mathrm{gap}}}{k} \tag{S3}$$

$$R_3 = \frac{\delta_{\mathrm{PS}}}{k_{\mathrm{PS}}} \tag{S4}$$

$$R = (R_1 + R_2) \parallel R_3 \tag{S5}$$

Here, R is the total parasitic thermal resistor, which is modelled as the parallel resistance between $(R_1 + R_2)$ and R_3. The thermal resistor of the reflector is neglected due to its high thermal conductivity of aluminium, k_{Al} = 237 W/(m-K), and low thickness (0.3 mm).

The key here is to estimate the convection coefficient on the PE cover. We examine two limits: natural convection without wind and forced convection with maximal wind velocity during the experiment. The former represents the scenario with the concentrator, while the latter represents the scenario without the concentrator. In one limit, the natural convection coefficient from Eq. (8.39) of Ref. [5] is

$$h_{\text{natural-convection}} = 0.82 Ra_d^{\frac{1}{5}} Pr^{0.034} \frac{k}{d} \tag{S6}$$

where $Ra_d \equiv \frac{gd^3}{vD}\frac{(T_{\text{amb}}-T_{\text{PE}})}{T_{\text{amb}}}$ is the Rayleigh number with the diameter, d, of the circular emitter, in which g is the gravitational constant. Pr $\equiv v/D$ is the Prandtl number, and k, v and D are thermal conductivity, kinematic viscosity and thermal diffusivity of the surrounding air, respectively. In the other limit, the forced convection coefficient from Eq. (6.68) of Ref. [5] is

$$h_{\text{forced-convection}} = 0.664 Re_d^{\frac{1}{2}} Pr^{\frac{1}{3}} \frac{k}{d} \tag{S7}$$

where $Re_d \equiv u_{\text{wind}} d/v$ is Reynold number, in which u_{wind} is the wind speed.

Using equations (S1)-(S7), we estimate the upper and lower bounds of R to be $R_{\max}$ = 0.54 (m^2-K)/W and $R_{\min}$ = 0.246 (m^2-K)/W, respectively. Correspondingly, the lower and upper bounds of the parasitic heat transfer coefficient are $h_{\min}$ = 1.9 W/(m^2-K) and $h_{\max}$ = 4.1 W/(m^2-K), which correspond to the lower and upper bounds of the shaded area in Fig. 5 of the main text.

The parameters we used are the maximum wind speed, $u_{\text{wind}} = 3.9$ m/s, during the measurement period; the physical properties of air (300K): $D = 2.21\times10^{-5}$ m^2/s, $v = 1.58\times10^{-5}$ m^2/s, $k = 2.26\times10^{-2}$ Wm^{-2}K^{-1}. Other parameters include $g = 9.8$ m/s^2, $d = 0.075$ m, $L_{\text{gap}} = 0.005$ m, $\delta_{\text{PS}} = 0.1$ m and $k_{\text{PS}} = 0.035$ W/(m-K). In addition, according to experimental measurements, we use $\frac{(T_{\text{amb}}-T_{\text{PE}})}{T_{\text{amb}}} = 1\%$ to estimate the Rayleigh number.

Supplementary Note S2: Daytime radiative cooling at varying haze concentration

Figure S3 illustrates passive daytime radiative cooling (PDRC) at varying concentrations of haze ($PM_{2.5}$). There are two features worth noting. Firstly, as haze concentration increases, PDRC performance, including radiative cooling power (P_{cool}) and temperature reduction (ΔT), first increases and then decreases. This trend results from the competing effect between the enhancement of sunlight scattering and the diminishment of the atmospheric window with an increase in haze concentration. At low haze concentration, the decrease of solar power surpasses the increase of atmospheric radiation power, resulting in an increase in both P_{cool} and ΔT with increasing haze concentration. At high haze concentration, the effect of sunlight scattering approaches saturation, and the enhancement of atmospheric power becomes predominant, leading to a decrease in both P_{cool} and ΔT with increasing haze concentration. The other feature is that the thin emitter (20 μm) outperforms the thicker emitter (80 μm) at low haze concentration, whereas the thicker emitter has superior performance at high haze concentrations. That is because an emitter with higher solar reflectivity is favored in a clear sky where solar radiation power substantially exceeds the atmospheric radiation power. The thick emitter with greater thermal radiation emissions is advantageous in a haze-polluted environment, where sunlight is significantly scattered while the atmospheric window is slightly diminished.

Supplementary Note S3: Analysis of various components of the power as a function of the cooler thickness

Taking PDMS/Ag as an example, we quantify the energy fluxes of a radiative cooler with different thicknesses of the transparent thermal emitter (PDMS) in both a clear atmosphere (Fig. S7a) and a haze-polluted atmosphere (Fig. S7b). Other atmospheric conditions are an ambient temperature of 10 °C and a relative humidity of 40% as a typical scenario. For clarity, we define a net MIR power:

$$P_{\mathrm{net-MIR}} = P_{\mathrm{rad}} - P_{\mathrm{atm}} \tag{S8}$$

As the emitter thickness (t) increases, the thermal emissivity, as shown in Figs. 4a-b of the main text, increases. Consequently, both the solar absorption power (P_{solar}) and the net MIR power ($P_{\mathrm{net\text{-}MIR}}$) decrease. The increase of $P_{\mathrm{net\text{-}MIR}}$ is fast initially and then slows down, while the increase of P_{solar} remains relatively stable. As a result, the net cooling power, $P_{\mathrm{cool}} = P_{\mathrm{net\text{-}MIR}} - P_{\mathrm{solar}}$, first increases and then decreases, regardless of weather conditions. In comparison to a clear atmosphere, the presence of haze results in a decline in both P_{solar} and $P_{\mathrm{net\text{-}MIR}}$, while the decrease in P_{solar} exceeds that in $P_{\mathrm{net\text{-}MIR}}$. Therefore, the optimal emitter thickness (t_{opt}) under haze pollution is greater than that in a clear atmosphere.

Supplementary Note S4: Analysis of scattering efficiency of haze particles

The scattering efficiency, E_s, is the ratio of the scattering cross section to the actual geometric projected area of the particle normal to the incident beam. E_s of particles exhibits a strong dependence on particle size relative to the wavelength of incident light [6]. The particles of around 10 nm and 60 nm comprise 75% and 15% of aerosols, respectively [1]. In Fig. S9a, we calculate the scattering efficiency of these two particles. Furthermore, we calculate E_s of haze particles (Fig. S9b) by the weighted average of the scattering efficiencies of high-concentration particles (D = 10 nm and 60 nm) according to their concentration ratios [1]. As a result, haze particles primarily scatter solar irradiance while slightly impacting thermal radiation. For simplification, we use the average optical constants of carbon and silica as the effective optical constants of haze particles.

Supplementary Note S5: Detailed analysis of the experimental results in Fig. 4c

In this note, we first argue theoretically (Fig. S15 and Table S1) that the haze and the water vapor are the two dominant factors to take into account the contrast in Fig. 4c. Then we rule out the water vapor with additional experiments (Fig. S14).

To identify the primary factor influencing the performance disparity in Fig. 4c, we examine the contributions of all potential variables. The performance of passive daytime radiative cooling (PDRC) is determined by solar irradiance, G_{total}, and the atmospheric radiation, E_{atm}, both received at the ground surface. We will now analyze the factors influencing G_{total} and E_{atm}, respectively.

G_{total} is subject to variations due to changes in the extraterrestrial radiation, G_{on}, and fluctuations of atmospheric compositions [7]. Two sources of variation in G_{on} should be considered. The first is the variation in the radiation emitted by the sun, which is at a rate of approximately 0.02% per year, so the energy emitted by the sun can be considered to be fixed. The other is the variation of the earth-sun distance. We use equation (1.4.1b) of Ref. [7] to estimate the dependence of G_{on} on the n-th day of the year:

$$G_{\text{on}} = G_{\text{sc}}(1.00011 + 0.034221\cos B + 0.00128\sin B + 0.000719\cos 2B + 0.000077\sin 2B) \tag{S9}$$

where $G_{\text{sc}} = 1367$ W/m^2 is the solar constant, and B is given by

$$B = (n-1)\frac{360}{365} \tag{S10}$$

The resulting variance in solar irradiance, ΔG_{total}, caused by the sun-earth distance change from March 25 to March 29 is estimated to be -2 W/m^2.

Next, we consider the fluctuations of atmospheric composition concentrations. The atmosphere is usually divided into three groups: dry air molecules, water vapor, and aerosols. The variations

of both G_{total} and E_{atm} predominantly depend on the concentration changes of aerosols, water vapor (H_2O), ozone (O_3), and carbon dioxide (CO_2) [8,9]. Figure S15 and Table S1 illustrate the calculated contributions of concentration changes in individual atmospheric components, from March 25 to March 29, to the fluctuations in solar irradiance, ΔG_{total}, and atmospheric radiation, ΔE_{atm}, using Modtran [9]. Figure S15 and Table S1 conclude that the haze dominates ΔG_{total} while the water vapor dominates ΔE_{atm}.

Finally, we rule out the water vapor experimentally. At a relative humidity (RH) of 35% (left panel of Fig. 4c; Fig. S14c), the thinner cooler performs at a lower temperature than the thicker cooler; conversely, at RH = 50% (right panel of Fig. 4c; Fig. S14b), the thicker cooler exhibits a lower temperature. However, as shown in Fig. S14(d), the thinner cooler maintains a lower temperature than the thicker cooler at a higher humidity of RH = 66%. Considering that the ambient temperatures (T_{amb}) during these three days are nearly identical, RH predominates the water vapour content in the surrounding environment. Therefore, the water vapour is not the element causing the performance discrepancy illustrated in Fig. 4c of the main text.

Supplementary References

1. Zhu, Y. Modelling analysis of spatiotemporal variation and source of atmospheric particle number concentration in typical cities of China. Doctoral dissertation, Nanjing University of Information Science and Technology, 2022.
2. Suichi, T.; Ishikawa, A.; Hayashi, Y.; Tsuruta, K. Performance limit of daytime radiative cooling in warm humid environment. *AIP Advances* **2018,** *8*, 055124.
3. Dong, M.; Chen, N.; Zhao, X.; Fan, S.; Chen, Z. Nighttime radiative cooling in hot and humid climates. *Optics Express* **2019,** *27*, 31587-31598.
4. China Meteorological Data Service Centre. http://data.cma.cn.
5. Lienhard, J. H.; Lienhard, J. H. A Heat Transfer Textbook, 4th ed.; Phlogiston Press: Cambridge, 2011.
6. Siegel, R.; Howell, J. R. Thermal Radiation Heat Transfer, 3rd ed.; Taylor & Francis: Washington, 1992.
7. Duffie, J. A.; Beckman, W. A. Solar Engineering of Thermal Processes, 4th ed.; John Wiley & Sons, Inc.: Hoboken, 2013.
8. Iqbal, M. An Introduction to Solar Radiation, Academic: Toronto, 1983.
9. Shen, S. S.; Berk, A.; Lewis, P. E.; Anderson, G. P.; Acharya, P. K.; Bernstein, L. S.; Muratov, L.; Lee, J.; Fox, M.; Adler-Golden, S. M.; Chetwynd, J. J. H.; Hoke, M. L.; Lockwood, R. B.; Gardner, J. A.; Cooley, T. W.; Borel, C. C.; Lewis, P. E.; Shettle, E. P., MODTRAN™ 5: 2006 update. In *Conference on Algorithms and Technologies for Multispectral, Hyperspectral, and Ultraspectral Imagery XII*, Shen, S. S.; Lewis, P. E., Eds. SPIE: 2006; Vol. 6233, p 62331F.
